# A Toolbox for Organelle Mechanobiology Research—Current Needs and Challenges

**DOI:** 10.3390/mi10080538

**Published:** 2019-08-16

**Authors:** Qian Feng, Sung Sik Lee, Benoît Kornmann

**Affiliations:** 1 Institute of Biochemistry, ETH Zurich, 8093 Zurich, Switzerland; 2 Institute of Molecular Health Sciences, ETH Zurich, 8093 Zurich, Switzerland; 3 Scientific Center for Optical and Electron Microscopy (ScopeM), ETH Zurich, 8093 Zurich, Switzerland; 4 Department of Biochemistry, University of Oxford, Oxford OX1 3QU, UK

**Keywords:** mechanobiology, mitochondria, organelles, microparticles, microfluidics, BioOptics, live-cell imaging

## Abstract

Mechanobiology studies from the last decades have brought significant insights into many domains of biological research, from development to cellular signaling. However, mechano-regulation of subcellular components, especially membranous organelles, are only beginning to be unraveled. In this paper, we take mitochondrial mechanobiology as an example to discuss recent advances and current technical challenges in this field. In addition, we discuss the needs for future toolbox development for mechanobiological research of intracellular organelles.

## 1. Introduction

Mechanobiology is the study of the interaction between mechanical stimuli and biological systems. Cells in the body are subjected to diverse mechanical stresses, including shear stress by blood flow, tensional stress due to muscle stretching, compression by the skeleton and more [1]. Past decades have seen tremendous interests and advances in this field, revealing mechano-regulation of an increasing number of biological processes [2]. Together with genetic and biochemical control, physical forces regulate cellular behavior during development, homeostasis and tissue regeneration, through signaling as well as gene expression programs.

Major efforts, thus far, have revealed how cells respond to changes in stiffness of the extracellular matrix, how cell surface receptors and mechano-sensitive channels are regulated by tension and shear stress on the plasma membrane, and how the cytoskeletal system senses and reacts to compressive forces [2,3,4,5]. However, mechano-sensing and -response are not limited to the plasma membrane and the cytoskeleton. The intracellular space is a dense and intricate environment. Organelles and proteinaceous complexes of all shapes are compressed into a minimal space; yet, they have to navigate around each other while engaged in dynamic, directed trafficking activities and morphological changes. How organelles deal with the resulting mechanical stress during homeostasis, and upon extracellular mechano-challenge, is only starting to be uncovered. The nucleus, the largest and bulkiest organelle in the cell, is normally protected by cytoskeletal elements, such as the Linker of Nucleoskeleton and Cytoskeleton (LINC) complex and actin-based structures around the nucleus [6]. However, upon severe mechanical compression of the cells, for instance when immune or cancer cells migrate through narrow openings of merely 1–2 μm, severe actin polymerization transiently forces open the nuclear membrane to allow cell passage, after which neat biochemical programs are called upon to restore nuclear membrane integrity and repair any DNA damage acquired during the brief nuclear membrane breakage [7,8,9]. Recent studies from our group and others showed that mitochondria, another crucial organelle that adopts complex and dynamic morphologies, can sense mechanical stress, and actively undergo biochemically regulated fission as a result [10,11,12]. How other organelles in the cell cope with mechanical stimulation is largely unknown. 

As for any emerging field, novel tools and novel applications of existing tools are being developed at a rapid pace for mechanobiological studies on the organismal, tissue, cellular, and molecular levels [13,14,15,16]. Microfluidic devices with controllable fluid flow have been instrumental in studying shear stress response by cultured cells [17,18,19]. Stretchable and/or bendable culture substrates have been successfully used to study cellular adaptation to stretching [20,21]. Controllable microfluidic trapping devices were recently used to study cell surface receptor signaling upon compressive forces [5]. Our group recently re-purposed atomic force microscope (AFM) to exert forces on individual mitochondrial tubules in live cells [10]. Nonetheless, the toolbox for intracellular mechano-manipulation, both qualitatively and quantitatively, remains limited. 

In this article, we will focus on recent developments on the mechanobiology of a crucial organelle, the mitochondrion. We discuss the current understanding and technical challenges in this field, comment on the existing tools, and recommend future developments in the engineering domain for this exciting research field. 

## 2. Mechanobiology of Mitochondria

### 2.1. Mitochondrial Function and Response to Mechanical Stimulation

**Figure 1:**
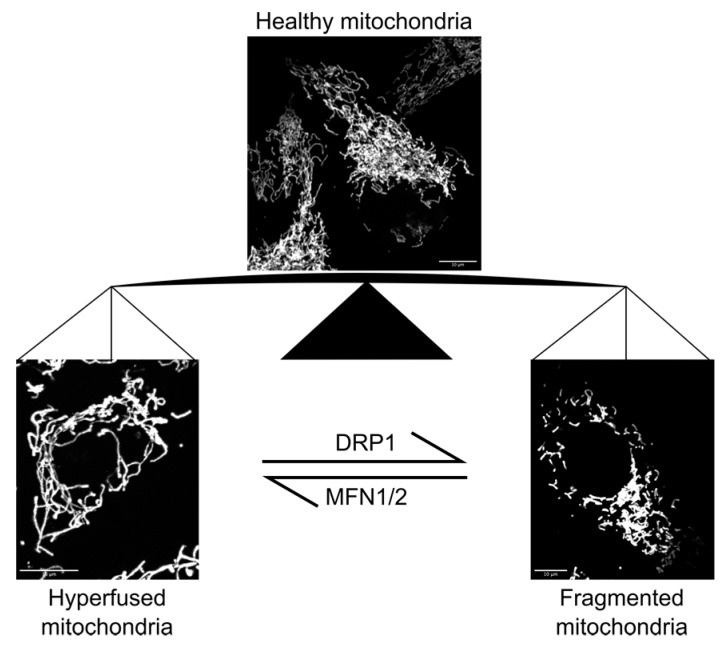
Mitochondrial morphology in human cells. Mitochondrial network in human osteosarcoma (U2OS) cells were visualized by expressing a mitochondria-targeted fluorescent protein [10] (top middle). Dynamin-related protein 1 (DRP1) depletion (bottom left) or overexpression (bottom right) resulted in hyperfused or fragmented mitochondrial morphologies, respectively [10]. Cells were imaged using an oil immersion objective (UPlanSApo, Olympus; Magnification 100×, NA = 1.4) on a DeltaVision microscope system coupled to a sCMOS camera (pco.edge 5.5, PCO). Depletion of mitofusin 1 and 2 (MFN1/2) has the opposite effect on mitochondrial morphology as DRP1 depletion [30,31] (not shown).

Mitochondria are responsible for the majority of the ATP produced in eukaryotic cells, and are often referred to as the powerhouses of the cell. In addition, mitochondria provide a signaling platform for cellular homeostasis, stress and immune response, cell cycle control and cell death [22,23,24]. In many cell types of higher animals, mitochondria are long inter-connected tubules, collectively forming a highly branched network. The morphology of the mitochondrial network is continually modified by dynamic events of fission (division) and fusion (Figure 1). Research from the last decades has shown that mitochondrial morphology, and the dynamic processes that govern it, are essential for mitochondrial function. Fission and fusion deficiencies have been associated with a variety of pathophysiological conditions, from diabetes, cancer development, neurodegenerative diseases to aging [25,26,27,28,29]. 

**Figure 2:**
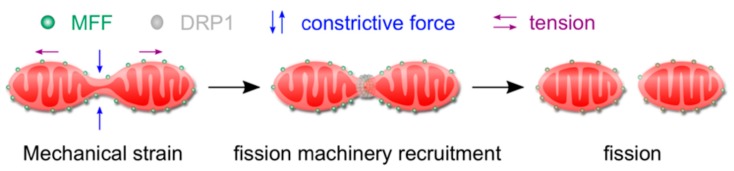
Schematic illustration of MFF-mediated force-induced mitochondrial fission. Mechanical stresses, including constrictive force (blue arrows) and tension (purple arrows) can cause local constrictions on mitochondrial tubules and increase the membrane tension at the constriction sites. MFF (green), an OMM protein, has an intrinsic preference for thinner tubules and, thus, concentrates at the constricted sections, effectively making MFF a mechano-sensor. Mechano-sensing is then translated to fission due to interactions between MFF and DRP1 (gray), the fission dynamin-related protein, which further constricts the tubule, eventually leading to membrane scission. Figure and figure legend are re-produced from Journal of Cell Science [6] with permission.

The molecular machineries that execute fission and fusion have been well described [30,31], however, the triggers that determine when and where in the cytoplasm these processes ought to take place remained long elusive. Recently, our group and others showed that mitochondria are mechano-sensitive, and that mechanical stress alone is sufficient to trigger mitochondrial fission. Mechano-fission is mediated by an integral membrane protein on the outer mitochondrial membrane (OMM), namely mitochondrial fission factor (MFF), which senses mechanically constricted sites on mitochondrial tubules thanks to its intrinsic propensity to localize to sites of reduced diameter. MFF oligomerization, in turn, recruits the fission dynamin protein Dynamin-Related Protein 1 (DRP1) to further constrict the membrane tubule, eventually leading to membrane scission (Figure 2). 

### 2.2. Current Challenges in Studying Mechanobiology of Mitochondria and Other Intracellular Organelles

**Figure 3:**
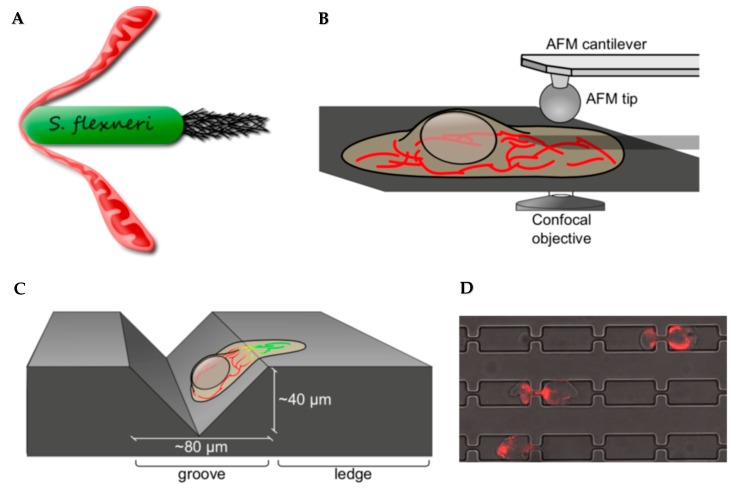
Methods to mechanically challenge mitochondria in live cells. (**A**) Motile intracellular bacterium, *Shigella flexneri*, was used to infect U2OS cells in culture. Shigella forms actin comet tails by polymerizing host actin at one pole, and thrusts within the cytoplasm of the infected host cell, thereby hitting and pulling on mitochondria that lay across their path. (**B**) Atomic force microscope (AFM) was used to transmit set amount force to single mitochondrial tubules in live U2OS cells. (**C**) Cell spreading on uneven surfaces, such as across the edge on a gramophone record, can induce constriction of the cytoplasm along the edge, thereby constricting mitochondria therein. (**D**) Cell migration through narrow constriction sites force cells to deform at the constriction sites, thereby mechanically stress the mitochondria therein. Human monocyte derived cells (THP-1) cells expressing a mitochondria-targeted DsRed fluorescent protein (red) were seeded on one side of the microfabricated channels. A serum gradient is given to promote cell migration through the channels towards the other side. Panels A–C are re-produced from Journal of Cell Science [6] with permission.

The experimental quest to study the mechanobiology of mitochondria was, and remains, technically challenging due to the lack of standard tools. In cells, mitochondria are extensively tethered to other organelles and cytoskeletal components, and are, therefore, constantly under tension. Purified mitochondria are free in solution, and tend to adopt a globular shape, making them a less suitable model for studying the biophysical properties of mitochondria in vivo. To mechanically stimulate mitochondria in live cells, we re-purposed existing tools including: (1) using AFM as a tapping device to exert force on mitochondrial tubules, (2) taking advantage of a motile intracellular bacterium, *Shigella flexneri*, as microbiological bullets to physically challenge mitochondria, and (3) growing adherent cells on the uneven surface of gramophone records, where cell spreading, on its own, constricted cytoplasmic contents including mitochondria (Figure 3A–C). Each method was uniquely valuable and limited, as discussed here below, and it was only the combination of these unconventional approaches, together with biochemical tools, that allowed us to draw the conclusions of the study [10].

AFM-mediated force application is low-throughput, because one must target single mitochondrial tubules in order to obtain clearly resolved images by simultaneous microscopy to assess the mechano-response. Furthermore, though AFM can precisely transmit a set amount of force to the target, most of the force is absorbed by the cortical actin networks, making it impossible to assess the amount of force eventually inflicted onto mitochondrial membranes and whether it is within physiological range. Both intracellular shigella infection as well as cell spreading on gramophone records exert biologically relevant amount of forces on mitochondria, but have other limitations of their own. Shigella intracellular motility is fueled by actin polymerization at one polar end of the bacterium, and thus, entirely unpredictable, leave alone controllable. Hours of live-cell imaging are necessary to identify few events of interest. In addition, late in the infectious life cycle of shigella, the host cell undergoes apoptosis due to extensive biochemical and biophysical damage by the bacteria. Thus, only events recorded in the very early stage of infection are relevant to mechano-studies. Gramophone records consistently constrict cytoplasm of cells at its sharp edges, but its nontransparent nature significantly limit the resolution of the microscopy images, making the analysis of protein recruitment difficult. Moreover, because all of the methods above actually stimulate mitochondria through other cellular materials, the involvement of these factors need to be additionally addressed using classical biochemical tools. Similarly, to study the role of tension in triggering mitochondrial fission, Carlini and colleagues relied heavily on mathematical and biophysical calculations [11]. Clearly, the ideal toolset(s) for studying mechanobiology of the subcellular organelles are, yet, to be perfected.

### 2.3. Exciting Questions to Answer in Mitochondria Mechanobiology, and the Tools We Will Need

The discovery of the novel phenomenon mitochondrial mechano-fission opens exciting avenues in mechanobiological research of this organelle. How much pressure is required to significantly constrict mitochondrial tubules to allow for MFF enrichment (See Section 2.1)? What is the elasticity of the OMM—commonly considered relatively fluid—and the Inner Mitochondrial Membrane (IMM)—considered rigid due to dense packing of proteins and cristae? 

Another important question in mitochondrial mechanobiology concerns the biological function of mechano-sensing by this organelle. Mechano-fission leads to mitochondrial division at constricted sites. But how frequent do such constrictions occur in vivo? In other words, is mechano-fission part of the regulatory mechanism to maintain cellular homeostasis, or is it a last-resort solution against extreme mechanical stress, before the tubule physically tears and potentially releases apoptotic and proinflammatory factors? Furthermore, can other membranous organelles trigger mechano-responses by mitochondria, and if so, what does it mean for intracellular organelle-organelle communication?

Research aimed at uncovering the molecular mechanisms of organelle mechano-sensing and -response call for the development of both intracellular mechano-stimulators as well as (membrane-integrated) mechano-reporters. To directly exert mechano-stress onto the target organelle in live cells, the ideal mechano-stimulator should be deliverable (by biochemical or biophysical methods) in a non-invasive manner. Additionally, the mechano-stimulator, and the applicable mechanism to control its movement in cells, should be compatible with microscopy methods (See Section 3 for further discussion on microscopy methods) so that the mechano-response of the target organelles could be simultaneously recorded. Solutions partially fulfilling these criteria have been reported (See Section 3), and may lay the foundation for future approaches. 

Functional assays aimed at studying the mechano-response of organelles mostly involve traditional biochemical and cell biological methods to study, for instance, protein recruitment and regulation of signaling cascades. In addition, microfabricated channels combined with microfluidic devices have been instrumental in studying the mechano-response of whole cells. Particularly, micro-channels containing constriction sites were developed to study cellular [32], and later nuclear [7,8] mechano-responses during cell migration as a model for tissue infiltration and cancer metastasis in vivo. Using slightly modified micro-channels—with even narrower constriction sites—we attempted to study the mitochondrial response during cell squeezing while they migrate through the constrictions (Figure 3D). Constriction sites of 1 µm or wider did not infer significant mechanical stress on mitochondria. Precisely manufacturing structures narrower than 1 µm would require advanced fabrication methods such as electron beam lithography and reactive-ion etching. 

## 3. Future Engineering Tools for Studying Mechanobiology of Mitochondria and Other Intracellular Organelles

### 3.1. External Field Control for Intracellular Mechano-Stimulation

Intracellular mechanobiological research calls for tools that can directly deliver a mechanical stimulus onto the organelle, without affecting the plasma membrane or other cytoplasmic elements. In this regard, methods utilizing external field control, such as magnetic, optical or acoustic fields, hold great potential. Magnetic tweezers manipulate paramagnetic beads using a gradient magnetic field [33,34,35]. It is possible to control the magnetic force by varying the distance between the magnets and the magnetic beads or by changing magnetization orientation. Such magnetic beads can be internalized by cells or delivered into the cytoplasm by microinjection, and have been used to measure physical properties (e.g., viscoelasticity and particle motile activity) of the cytoplasm since decades [36,37]. Magnetic control has also been explored to study various biological processes at the cell surface in more recent years [38,39]. Lately, a magneto-genetic approach was used to study intracellular signaling in live cells. Magnetic particles were functionalized with a HaloTag ligand and injected into the cytoplasm of cells, where HaloTag-fusion protein of interest could be captured and manipulated using the external magnetic field [40]. Indeed, fluorescently labeled, magnetic nanoparticles may prove instrumental in studying mitochondrial mechanobiology. Once introduced into the cytoplasm of cells, particle motion could be controlled by the external magnetic field to mechanically stimulate mitochondrial tubules in a highly targeted manner. Mechanical force necessary to trigger a specific biological response of interest (e.g., MFF oligomerization) could, in turn, be calculated based on the strength of the magnetic field necessary to cause the said response. Similarly, optical [41,42] and acoustic [43,44] tweezers could make excellent alternatives for remote manipulation of intracellular particles to mechanically challenge cytoplasmic organelles. Further, remotely controllable micro-robotics that can *record* force encounter may hold a key for future developments of mechano-stimulators and -reporters. 

### 3.2. Detection of Forces/Tension on a Biological Membrane 

**Figure 4:**
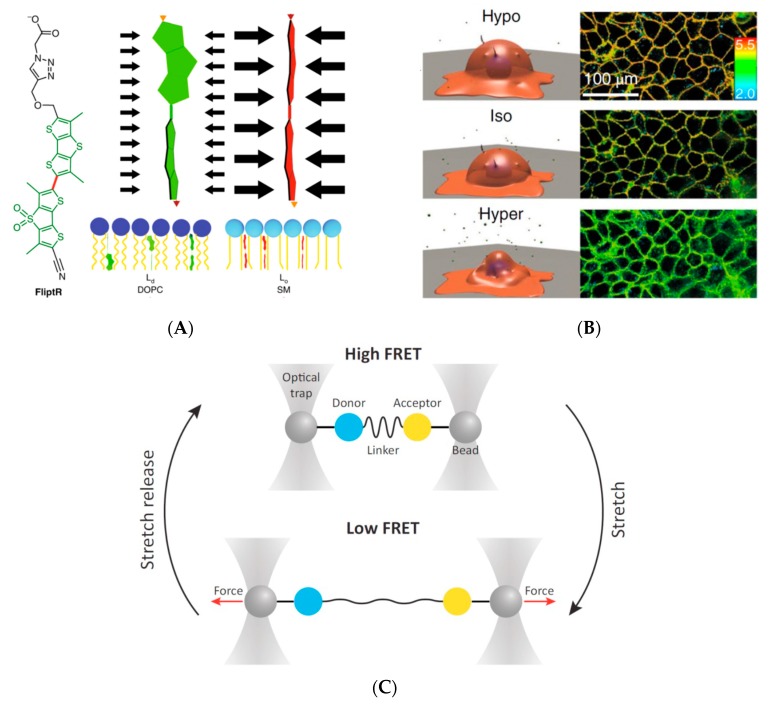
Fluorescent imaging based mechano-sensor. (**A**) An example of pressure-sensitive probe, FliptR. Pressure along the axis, for instance from lipid packing, can planarize the two fluorescent groups, leading to changes in excitation maxima and fluorescence lifetime. Figure and caption reproduced from Spring Nature [46] with permission. (**B**) Response of FliptR (see A) fluorescence lifetime to osmotic shocks on cells. FLIM images of MDCK in isoosmotic buffer, and after hyper- or hypoosmotic shocks. Figure reproduced from Spring Nature [46] with permission. (**C**) Schematic illustration of force-FRET sensor. The purified tension sensor module, containing donor and acceptor fluorophores as well as the mechanosensitive linker peptide, is attached to two microbeads; a dual optical tweezer setup is used to apply pico-newton (pN) forces. The resulting force-extension correlations can be used to calculate the force-FRET response. Figure reproduced from Elsevier [47] with permission.

More relevant than measuring how much force is applied, is measuring how much force is received on the surface of a given target organelle. Although direct quantification of forces/tension on a biological membrane in live cells is also more challenging, some successful attempts have been reported. Biological membranes are fluid-dynamic, and compressive force or tension can change the lipid-packing density. Based on this property, planarizable push-pull fluorescent probes have been developed that change emission spectra dependent on the degree of lipid packing, and have shown great potential on giant unilamellar vesicles and cells [45,46] (Figure 4A,B). 

Other mechano-sensors include various fluorescence energy transfer (FRET)-based systems [48,49]. Two fluorophores capable of undergoing efficient energy transfer are connected by a spring-like linker—made of peptides or nucleic acids—that stretches in response to mechanical forces (Figure 4C). A decrease in FRET signal, thus, reflects a greater distance between the two fluorophores. All these probes have been demonstrated to function to varying degrees at the plasma membrane, but still require optimization for cytoplasmic use. Importantly, while these sensors can report tension, no design can, yet, successfully report compressive forces.

### 3.3. Assessing Biophysical Properties of Intracellular Organelles by Quantitative Phase Imaging (QPI)

**Figure 5:**
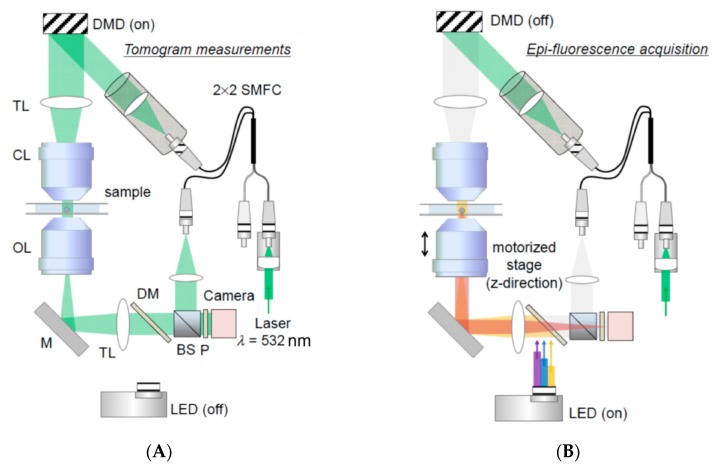

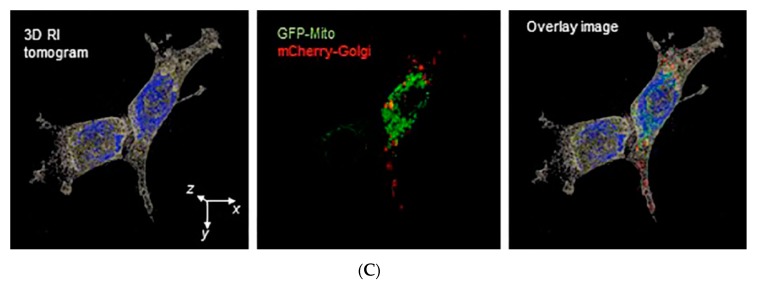
Quantitative Phage Imaging. The combined optical setup for optical diffraction tomography (ODT, **A**) and 3D epifluorescence microscopy (**B**). DMD, digital micromirror device; TL, tube lens; CL, condenser lens; OL, objective lens; M, mirror; DM, dichroic mirror; BS, beam splitter; P, polarizer; LED, light emitting diode. Figure reproduced from Biomedical Optics Express under CC BY License [56] with permission. (**C**) Demonstration of applicability of ODT and multimodal approach combining ODT and fluorescence. NIH-3T3 cell images through ODT and 3D FL (GFP-Mito and mCherry-Golgi). Both images are obtained by a commercialized ODT setup (HT-2H, Tomocube, Inc., Daejeon, South Korea). Figure reproduced from Yale Journal of Biology and Medicine [57] with permission.

Another challenge in intracellular mechanobiology is the measurement of biophysical properties, such as rigidity and density, of organelles in their native environment. These properties not only teach us about the basic features of the organelles, changes in such characteristics can also serve as first indications of biological changes such as lipid packing and protein accumulation. To this end, a group of recently developed methods, collectively known as quantitative phase imaging (QPI), holds great potential (Figure 5). QPI is an interferometric microscopy technique that measures the optical phase delay induced by refractive index (RI) difference between a sample and the medium. RI, in turn, can be correlated to important biophysical characteristics including mechanical, electrical and optical properties [50]. 

While still in early stage of development, QPI applications have already been used to report biophysical changes on the cellular level. Park and colleagues used diffraction phase microscopy to extract mechanical changes of red blood cells during shape transition, and successfully differentiated different stages based on QPI measurements [51]. Others reported a reversed correlation between the disorder strength and cell stiffness, and demonstrated differential changes in stiffness upon shear stress in different cell lines [52]. Zooming into the subcellular environment, a recent report utilized refractive index tomography to generate a 3D reconstruction of the RI map of live cells, where the nucleus, nucleoli, and some cytoplasmic entities could be simultaneously assessed [53], laying a foundation for future multi-organelle analysis upon stimulation or stress. Efforts are also currently being undertaken to precisely measure the RI of purified subcellular organelles, including mitochondria [54], to facilitate the generation of RI standards for future studies. More recently, an improved QPI microscopy method was reported, which enabled visualization of mitochondria trafficking on microtubules in a label-free fashion with superior temporal resolutions (essentially only limited by the camera frame rate) [55]. 

### 3.4. Assessing (Bio-)Chemical Properties of Intracellular Organelles by Raman Spectroscopy-Based Methods

Besides the biophysical responses of organelles upon mechanical stimulation, it is naturally also crucial to measure the biochemical changes such as protein modifications and recruitment, as well as lipid composition changes. Traditionally cell biologists have relied largely on live-cell fluorescence microscopy for this purpose, as it provides the desired organelle specificity thanks to genetic coding (e.g., fluorescent proteins fused to a mitochondria-targeting signal peptide) or chemical modifications (e.g., membrane potential-sensitive probes for mitochondria). However, fluorescence microscopy methods are inevitably limited by the number of fluorophores that are simultaneously resolvable or can be acquired sequentially without too much time lapse in between. 

**Figure 6:**
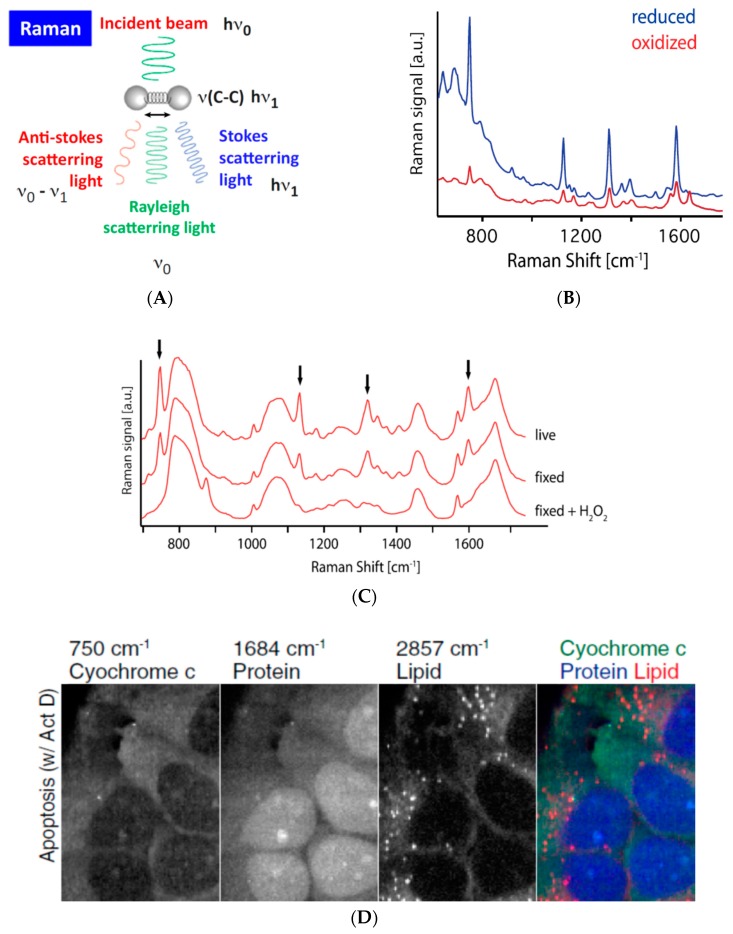
Raman microscopy. (**A**) Schematic illustration of Raman scattering. (**B**) Raman spectra of a cytochrome c solution under the reduced and oxidized state. (**C**) Raman spectra of a selected area in a live HeLa cell, the same area after fixation by paraformaldehyde, and additional H_2_O_2_ treatment. The Raman signature peaks assigned to cytochrome c are indicated with black arrows. (**D**) Raman images of apoptotic HeLa cells at 750, 1684, 2857 cm^−1^, showing the distribution of cytochrome c, protein beta sheet, and lipid molecules, respectively. Panel A is reproduced from Elsevier [60] with permission, and B–D from PNAS [58] with permission.

Raman spectroscopy (referred to as Raman here below) may prove an excellent complementary approach as it potentially allows simultaneous, label-free detection of several biological entities while retaining the same 2D resolution of a classical confocal microscope (Figure 6). This method detects molecular vibration mode, which is sensitive to changes in the molecular structure and their surrounding environment. One single Raman spectroscopic scan (of an entire cell or multiple cells in the imaging field) can be used to mathematically extract information on individual elements therein by using the Raman spectra of purified elements as standards. As a proof-of-concept, Okada and colleagues applied Raman to demonstrate the change in oxidization status of recombinant cytochrome C upon ascorbic acid treat and the loss of mitochondrial localization of cytochrome C during apoptosis in live cells [58]. In another report the Raman spectra of yeast cells deficient in mitochondrial fission (ΔDnm1, ΔFis1) or fusion (ΔFzo1) could be distinguished from that of WT cells and each other [59], demonstrating the potential to use Raman scans to identify subpopulations of cells carrying genetic variations.

The Raman microscopy field is evolving at a rapid pace [61]. Surface Enhanced Raman Scattering (SERS) aims to achieve a better signal-to-noise ratio, while Coherent Anti-Stokes Raman scattering (CARS) and Stimulated Raman Scattering (SRS) reduce photo toxicity by using nonlinear Raman scattering techniques and near-infrared illumination. Raman microscopy can also be used in combination with Brillouin microscopy, which probes elastic properties of materials by using inelastic scattering of light, to obtain mechanical properties on the subcellular scale [62,63]. Meanwhile, advances in mathematical modelling continue to support Raman applications to achieve increasingly higher specificities (e.g., to differentiate subcellular organelles, or even subspecies of phospholipids, from one another). In addition, using fluorescently labeled samples as training sets, artificial intelligence- and machine learning-assisted approaches are being developed to advance Raman specificity even more, with the goal to detect chemical changes in a complex sample, such as a cell, in the future [64,65]. 

## 4. Concluding Remarks

Mechanobiology of subcellular organelles is just beginning to be tackled. Traditional tools commonly employed in mechanobiology on the organismal and cellular levels fall short in delivering direct mechano-stimulation within the cell. In addition, the detection of biophysical properties, and mechano-responses of subcellular organelles require novel methods. The challenges and desired functionalities of future tools described here are not unique to the mitochondrion, but applicable to all intracellular components, the mechano-properties of many of which are, to date, largely unknown. It is clear that successful tool development in this domain will require close collaborations between experts in microfabrication, nanorobotics, as well optics and sensor technologies; and above all, continuous dialogue between technology developers and potential users.
